# Functional assessment of high-grade ICA stenosis with duplex ultrasound and transcranial Doppler

**DOI:** 10.1111/j.1475-097X.2011.01118.x

**Published:** 2012-05

**Authors:** Helene Zachrisson, Marita Fouladiun, Christian Blomstrand, Jan Holm, Reinhard Volkmann

**Affiliations:** 1 Clinical Physiology, Department of Medical and Health Sciences, Linköping University, LinköpingSweden; 2 Clinical Physiology, Sahlgrenska AcademySweden; 3 Departments of SurgerySweden; 4 Neurology, Sahlgrenska Academy, GothenburgSweden

**Keywords:** carotid artery disease, collateral blood flow, duplex ultrasound, transcranial Doppler

## Abstract

**Background:**

Duplex ultrasound (DUS) has shown a >90% accuracy compared to angiography, concerning the degree of internal carotid artery (ICA) stenosis. However, uncertainty may occur in a severe stenosis, in which peak systolic velocity (PSV) may decrease owing to high flow resistance or high backward pressure. We investigated intracranial collateral flows using transcranial Doppler (TCD) to further evaluate the hemodynamic significance of high-grade ICA stenosis.

**Methods:**

In this retrospective study, 320 consecutive symptomatic patients were examined. The degree of ICA stenosis and collateral capacity in the circle of Willis was investigated by DUS and TCD. In addition, magnetic resonance angiography (MRA) was added in a subgroup of 204 patients. The criterion for hemodynamic significant ICA stenosis was established collateral flow.

**Results:**

In 91% of all symptomatic vessels (291 vessels), an ICA stenosis of ≥70% was found. Established collateral flow always indicated precerebral carotid artery disease of ≥70%. Furthermore, in 11% of the whole study material, collateral reserve capacity was found despite high-grade (≥70%) ICA stenosis. PSV in ICA <2·5 m s^−1^ was combined with established collateral flow and MRA stenosis of ≥70% in 9% (19 arterial systems). In 4%, doubt existed concerning the degree of stenosis after DUS.

**Conclusion:**

Transcranial Doppler helps to determine whether an ICA stenosis is of hemodynamic significance and to assess collateral patterns. Established collateral blood flow will help to identify patients with ≥70% (ECST) carotid artery disease. TCD might be of value when flow velocity criteria combined with plaque assessment by DUS are inclusive. Other diagnostic methods may also be considered.

## Introduction

Duplex ultrasound techniques have widely replaced preoperative carotid angiography in clinical routines. Internal carotid artery (ICA) disease can functionally be assessed by the measurement of peak systolic velocity (PSV) within the stenosis and then translated into terms of percentage diameter reduction (Rahma *et al.*, 2011). The diagnostic accuracy of duplex ultrasound (DUS) has been widely tested using angiography as a reference and shown to be more than 90% when performed by experienced sonographers (Zbornikova *et al.*, 1985; Jogestrand *et al.*, 2002; Rahma *et al.*, 2011).

In some cases, a high-grade stenosis may show maximal velocities lower than the used limit, not only as a result of increased resistance or turbulence (Spencer & Reid, 1979) in the stenotic area but also as a result of intracranial collateral compensation (Zachrisson *et al.*, 2001). In a previous study, we demonstrated that the collateral support of the ipsilateral middle cerebral artery (MCA) may increase poststenotic blood pressure in the ICA. This may reduce blood pressure gradients over the stenosis, resulting in a decrease in ICA flow (Zachrisson *et al.*, 2001). The measurement of relatively low ICA blood flow velocities, without adequate detection of plaque burden, might lead to an underestimation of the degree of stenosis and perhaps exclusion from considered surgical treatment.

Transcranial Doppler (TCD) can be used to assess collateral flows as well as signs of microembolia, which might be a possible indicator for carotid plaque instability (Zachrisson *et al.*, 2000a,b; Zuromskis *et al.*, 2008; Topakian *et al.*, 2011).

Exhausted cerebrovascular reserve capacity has been reported to increase the risk of stroke in asymptomatic patients (Engelhardt *et al.*, 2004). An evaluation of intracranial collateral compensations with TCD is therefore of interest and may help to assess ICA diameter reductions inducing collateral blood flow.

This ultrasound study of patients with symptomatic carotid stenosis aimed to correlate certain intracranial collateral blood flow patterns with different degrees of carotid artery disease. Our hypothesis was that functional high-grade carotid artery disease could be defined with higher diagnostic accuracy after additional TCD analysis of the collateral blood flow capacities to the ipsilateral MCA.

## Methods

### Patients

During a 4-year period, 320 consecutive patients (mean age 67, range 39–89 years, 34% women) with a history of amaurosis fugax, transitory ischaemic attacks or minor strokes were referred to our carotid centre because of diagnosed or suspected symptomatic carotid stenosis. All patients were retrospectively analysed concerning the grade of ICA stenosis with DUS.

**Figure 1:**
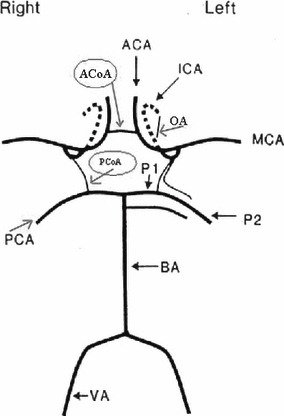
Circle of Willis, ACoA, Anterior communicating artery; PCoA, Posterior communicating artery; OA, Ophthalmic artery; ICA, Internal carotid artery; MCA, Middle cerebral artery; PCA, Posterior cerebral Artery; P1, proximal segment of posterior cerebral artery; P2, distal segment of posterior cerebral artery; VA, Vertebral artery; BA, Basilar artery.

All patients also performed a TCD, and all investigations were reviewed concerning collateral capacity (Fig. 1).

Magnetic resonance angiography (MRA) was performed in a subgroup of 204 patients, and the grade of ICA stenosis was reviewed.

### Duplex ultrasound

**Figure 2:**
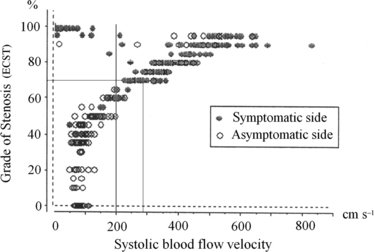
Bivariate scatter plot of the maximum systolic blood flow velocity (*V*_systmax_, cm s^−1^) and the estimated diameter reduction (% stenosis) within available data from 409 internal carotid arteries (ICA). In 8% (34 ICAs; 32 symptomatic and 2 asymptomatic), the stenosis was suspected to be tight owing to duplex observations of very small colour flow channels within the stenosis despite maximum systolic flow velocities of <2·0 m s^−1^. In these cases, transcranial Doppler showed signs of established collateral flow to the ipsilateral side.

The technique of DUS has been described previously (Strandness, 1990). Briefly, the degree of carotid artery disease was assessed with duplex ultrasound (5-MHz linear 2D transducer equipped with 5-MHz pulsed Doppler, Acuson XP; Acuson Corp., Mountain View, CA, USA). All major neck vessels, that is the common carotid artery (CCA), internal carotid artery (ICA) and external carotid artery (ECA) carotid arteries as well as the vertebral (VA) arteries, were investigated. The Doppler beam was angulated at 60° to the blood flow vector. The grade of ICA disease was functionally assessed with measurements of PSV within the ICA stenosis and translated into percentage diameter reductions (Fig. 2, ECST method) [Randomised trial of endarterectomy (European Carotid Surgery Trail, 1998)] based on comparisons between duplex ultrasound and selective carotid angiography (Zbornikova *et al.*, 1985). Our criteria are in agreement with results from a Swedish multicentre study comparing systolic and diastolic flow velocity data with carotid angiography (Jogestrand *et al.*, 2002).

### Transcranial Doppler

The circle of Willis was examined according to the study of Aaslid *et al.* (Aaslid *et al.*, 1982) and our own experience (Zachrisson *et al.*, 2000a,b; Jatuzis *et al.*, 2000) using a 3D TCD flow-mapping device (Transcan, EME, Überlingen, Germany) equipped with 2-MHz pulsed Doppler transducers. The middle, anterior and posterior cerebral arteries were investigated using the transtemporal approach, whereas the ophthalmic arteries (OA) were insonated with a pulsed 4-MHz transducer through the transorbital window. All blood flow velocity signals were analysed to determine direction and velocity of the blood flow. The collateral blood flow capacities in the anterior communicating arteries (ACoA) and posterior communicating arteries (PCoA) were also evaluated by CCA compression (comp-CCA) just above the clavicular bone (Zachrisson *et al.*, 2000a,b; Jatuzis *et al.*, 2000). In patients with clearly protruding heterogeneous CCA plaques or with signs of carotid dissection, comp-CCA was avoided.

We used a classification of various collateral flow alternatives as follows:

Collateral flow type 0, a*bsence of collateral flow*. Established collateral flow neither under baseline conditions nor during CCA compression.
Collateral flow type 1, *reserve flow capacity.* Detection of blood flow after CCA compression in any collateral pathway to the ipsilateral MCA.
Collateral flow type 2, *established collateral flow.* Detection of blood flow in any collateral pathway to the ipsilateral MCA, without CCA compression.
Collateral flow type 2, *established collateral flow.* From the ipsilateral to the contralateral MCA.

Table 1 shows these definitions of relevant collateral blood flow types through any assessable collateral pathway to the MCA. Figure 1 shows a schematic drawing of the circle of Willis indicating the collateral pathways. All TCD investigations were reviewed according to above-mentioned collateral flow alternatives.

**Table 1:** Definitions of collateral flow types to the relevant MCA. See also Methods of transcranial Doppler. See also the schematic drawing of circle of Willis (Fig 1).

Vessel	Type	Definition
MCA	0	Almost abolished MCA blood flow velocities upon ipsilateral comp- common communicating arteries (comp-CCA)
ACoA	0	Absence of blood flow in ACA-A1 upon ipsilateral comp-CCA
	1	Reversible blood flow in the ipsilateral ACA-A1 or flow acceleration in the contralateral ACA-A1 upon ipsilateral comp-CCA
	2	Reversed flow in ipsilateral ACA-A1, decreasing upon contralateral comp-CCA
	−2	Reversed flow in contralateral ACA-A1, decreasing upon ipsilateral comp-CCA
PCoA	0	Absence of blood flow increase in PCA-P1 upon ipsilateral comp-CCA
	1	Blood flow increase in ipsilateral PCA-P1 upon ipsilateral comp-CCA
	2	Increased blood flow velocities in ipsilateral PCA-P1 (*V*_max_ P1 > P2)
	−2	Reversed blood flow in PCA-P1 on relevant side
OA	2	Reversed flow in the OA

MCA, Middle cerebral artery; ACA-A1, proximal segment of the anterior cerebral artery; ACoA, Anterior communicating artery; PCA-P1, Proximal segment of the posterior cerebral artery; PCoA, Posterior communicating artery; PCA-P2, PCA segment distally to PCoA; OA, Ophthalmic artery; Comp-CCA, Proximal compression of the common carotid artery.

The collateral pathways are pointed out in the schematic drawing of the circle of Willis in Fig. 1.

### Magnetic resonance angiography

The results of MRA were reviewed in the case of ambiguous ultrasound outcome. The reason for the MRA was predominantly duplex findings of extensive plaque formations as well as unexpected low blood flow velocities, within ICA or technical reasons such as shadowing (calcified) plaques or tortuous vessels.

### Statistical analysis

Data were analysed by Student's unpaired tests using Statview® (version 5·01; SAS Institute Inc, Buckinghamshire, UK). Values are presented as mean ± SD. Statistical significance was set to *P*<0·05.

Distributions of observations are shown using descriptive statistics.

## Results

In the whole study group of 320 patients (320 symptomatic sides, 266 contralateral asymptomatic sides), the ultrasound degree of the ICA stenosis was available.

Five hundred eighty-six carotid bifurcations as well as the circle of Willis were investigated by DUS and TCD. Flow velocity data from the duplex ultrasound investigation were available in 409 arterial systems.

Missing collateral TCD flow data were attributable to acoustic difficulties to insonate the temporal bone as well as TCD investigation performed without CCA compression (8%, 54 sides).

**Figure 3:**
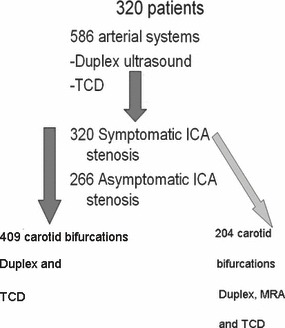
Flow diagram of the study material.

A subgroup of patients (204 carotid bifurcations) was completely investigated with MRA, DUS and TCD (See Fig. 3 flow diagram).

### Duplex outcome

In 64% (376 vessels), an ICA stenosis with diameter reductions of ≥70% was found. In 91% of all symptomatic vessels (291 vessels), an ICA stenosis of ≥70% was found. Thus, 85 ICA stenoses were asymptomatic.

Figure 2 shows the relationship between the maximum systolic blood flow velocity within the ICA stenosis (*N* = 409) and the estimated percentage diameter reduction (ECST method).

In 8% of the ICA stenoses (32 symptomatic and 2 asymptomatic), the stenosis was suspected to be very tight owing to the duplex observations of very small colour flow channels within the stenosis despite maximum systolic flow velocities of <2·0 m s^−1^. In these cases, TCD showed signs of established collateral flow and thus indicated high-grade ICA stenosis.

### Duplex and transcranial Doppler outcome

Tables 2 and 3 show the frequencies of the different collateral blood flow types in various grades of ICA disease. Established collateral blood flow to the relevant MCA was not observed in cases with <70% ICA stenosis, except in 12 vessels (3 symptomatic and 9 asymptomatic cases). In all these cases, a high-grade stenosis was found either within the CCA or in the brachiocephalic trunk (three cases) alternatively within the siphon (nine cases). Thus, an established collateral flow to the MCA always indicated precerebral vessel disease with ≥70% stenosis.

**Table 2:** Frequencies of collateral flow pattern types 0, 1, 2 and −2, respectively (in any of assessable collaterals, see Methods and Table 1) in various grades of symptomatic ICA disease.

Asymptomatic ICA stenosis [%]	Collateral flow types [*N*]			

	0	1	2	-2
<70	2	22	3	2
70–79	3	23	24	1
80–89	6	15	46	2
90–99	6	8	126	1
100	0	0	30	0
Σ: 320 patients (%)	17 (5·3)	68 (21·3)	229 (71·6)	6 (1·9)

The symptomatic side of totally 320 patients is investigated by transcranial Doppler and duplex. Collateral type 2 (established collateral flow to the relevant side) is only seen in internal carotid artery (ICA) stenosis of ≥70%, except in those cases with signs of high-grade arterial disease within the brachiocephalic trunk, common carotid artery or distal parts of the siphon (the cell representing these cases is marked in grey colour).

**Table 3:** Frequencies of collateral blood flow types in various grades of asymptomatic internal carotid artery (ICA) disease.

Symptomatic ICA stenosis [%]	Collateral flow types [*N*]			

	0	1	2	−2
<70	24	88	9	60
70–79	0	9	4	11
80–89	0	5	11	6
90–99	3	0	19	5
100	0	1	11	0
Σ: 266 patients (%)	27 (10·1)	103 (38·7)	54 (20·3)	82(30·8)

The asymptomatic side of totally 266 patients is investigated by transcranial Doppler and duplex. (for further explanations, see also Table 2).

Reserve collateral flow was seen in (61 cases) 10% of high-grade (≥70% stenosis) ICA stenosis. As also seen in Tables 2 and 3, reserve capacity could still be detected in the case of >90% ICA stenosis. Absence of collateral flow was seen in 18 cases (6%) of high-grade (≥70% stenosis) ICA stenosis.

### Duplex transcranial Doppler and magnetic resonance angiography outcome

In 9%, (19 ICAs), DUS showed significant plaque formations, but blood flow velocities were below 2·5 m s^−1^. In 11 of these, very tight (99%) ICA stenosis was diagnosed with low systolic blood flow velocities (<0·7 m s^−1^) and almost zero end diastolic blood flow velocities (string signs) as well as extensive plaque formations. In all these cases, MRA detected 99% ICA stenosis or occlusion.

In all the 11 ICAs with ‘string flow pattern’ and the 8 ICAs with systolic blood flow velocities below 2·5 m s^−1^, MRA showed signs of more than 70% ICA stenosis, and TCD showed established collateral flow. Thus, in 4%, doubt existed concerning the degree of stenosis after DUS.

The PSV showed insignificant differences in cases with established collateral flow compared to those with reserve capacity in high-grade (>70–99%) unilateral ICA stenosis (PSV 4·0 ± 1·9 m s^−1^ versus 3·6 ± 1·2 m s^−1^, p-value 0·4).

## Discussion

This study aimed to evaluate combined preoperative DUS and TCD diagnostics in patients with symptomatic carotid artery disease, by relating ICA blood flow pattern to different levels of intracranial collateral blood flow (Jatuzis *et al.*, 2000; Zachrisson *et al.*, 2001). We demonstrated that TCD helps to determine whether a stenosis defined to be high grade with DUS may also have hemodynamic significance. Patients with TCD signs of established collateral flow generally showed high-grade stenosis (>70%) both with DUS and with MRA. However, in up to 9% of the arterial systems, we found low PSV (<2·5 m s^−1^), established collateral flow and MRA stenosis ≥70%. This shows that some patients with relatively low ICA blood flow velocities, and perhaps without adequate detection of plaque burden, may be falsely underestimated concerning degree of stenosis. Interestingly, we also found that some high-grade stenosis could have maintained collateral reserve capacity.

Carotid endarterectomy (CEA) guidelines in symptomatic carotid artery stenosis are based on ECST and NASCET criteria with 70% or greater carotid stenosis as estimated from the angiogram [Randomised trial of endarterectomy (European Carotid Surgery Trail, 1998)]; Barnett *et al.*, 1998). Doppler criteria may however lead to underestimation in cases with decrease in maximal flow velocities caused by increased resistance or turbulence in the stenosis or by low flow caused by high distal collateral pressure (Spencer & Reid, 1979; Zachrisson *et al.*, 2001). Serial stenoses may also cause low flow and decrease stenotic flow velocity in the ICA.

In these patients, it seems important to compare DUS of ICA and different collateral patterns in the circle of Willis to evaluate the hemodynamic significance of the stenosis. Our results are in accordance with other reports stating that ≥70% carotid artery diameter reductions (ECST) can be detected by TCD (Byrd *et al.*, 1999; Christou *et al.*, 2001). We also found that all patients with sub-occlusive ICA considered for surgery had good collaterals as well as high stump pressures perioperatively (results of stump pressure nor shown), which, apart from the severeness of stenosis, might have been one explanation for extremely low flow velocities.

In a previous retrospective study, we found that diastolic flow velocities in severe carotid artery stenosis are inversely dependent on collateral back pressure in the distal part of ICA. High diastolic flow velocities and low pressures in the ICA during cross-clamp (stump pressures) characterized all patients with postoperative major stroke in our centre during a 9-year period (Zachrisson *et al.*, 2000a,b). In another prospective study, we demonstrated that the flow velocities within high-grade ICA stenosis were inversely correlated with the stump pressure (Zachrisson *et al.*, 2001).

The risk of both underestimation and overestimation of the degree of stenosis with MRA and DUS has been reported (Clevert *et al.*, 2006). Overestimation of the degree of stenosis with DUS may be explained by erroneously used angle corrections, for example using higher angles than 60° or the presence of compensatory increase in volume flow owing to contralateral severe stenosis/occlusion (van Everdingen *et al.*, 1998). Underestimation of the degree of stenosis with DUS is easily carried out as shown above, but also limited investigator experience and curved vessels may cause problems. Although there is no complete agreement as to which patients need surgery, it seems important that high-grade stenoses should not be missed as the risk of neurological symptoms may increase with increasing degree of stenosis. TCD might also be useful in the preoperative investigation before CEA, not only for assessing complementary information concerning degree of carotid stenosis but also to detect microembolic signals as well as intracranial collateral flow patterns (Kjällman *et al.*, 1995; Markus *et al.*, 2010; Topakian *et al.*, 2011).

Transcranial Doppler has been used to describe the hemodynamic significance of the ICA stenosis (Byrd *et al.*, 1999; Christou *et al.*, 2001), but earlier studies have not distinguished between patients with lack of intracranial collaterals, established collaterals and flow reserve capacity despite high-grade stenosis. The latter group might also be an interesting group, considering the fact that exhausted cerebrovascular reserve capacity has been reported to increase the risk of stroke in patients with asymptomatic carotid stenosis (Engelhardt *et al.*, 2004).

To evaluate the collateral circulation is also important concerning the aspects of the risk of hemodynamic neurological events owing to hypoperfusion (Moustafa *et al.*, 2010). Lack of collaterals might be of interest for surgical procedures (Kjällman *et al.*, 1995).

We also found even if established collateral flow always identified high-grade ICA stenosis, reserve capacity could still exist. One explanation for those unused collateral reserves is the existence of bilateral carotid disease in hemodynamic balance, balanced high-grade stenosis in external carotid artery (ECA) and ICA (detected when investigating the ophthalmic artery) or stenosis of the vertebrobasilar system. Data on vertebral arteries are not included in this study because of incomplete data. However, in this study, we defined existing collateral capacity in any collateral pathway; that is, even in case of a significant vertebral artery stenosis, inducible collateral flow in other collateral pathways might be possible.

In this study, we used TCD and evaluated the collateral capacities by means of CCA compression, which is commonly used for the testing of dynamic cerebral autoregulation (Moppett & Mahajan, 2004). Even by using transcranial colour-coded real-time sonography, it is not possible to assess collateral function without CCA compression (Nedelmann *et al.*, 2009). As long as CCA is compressed very proximally just above the clavicular, the risk is small (Jatuzis *et al.*, 2000). In this study, compression of the CCA did not elicit cerebral symptoms in any case. It had been reported that in 98% of symptomatic ICA disease, putting pressure on the CCA does not provoke additional microembolic signals despite a 30% probability of spontaneous microembolizations when investigated preoperatively by power M-mode TCD (Zuromskis *et al.*, 2008).

We have validated our results of CCA compression by perioperative measurements in a separate study. In no cases, the flow velocity decrease preoperatively was incomplete using the measurement during cross-clamp as a reference (Zachrisson *et al.*, 2000a,b).

## Limitations of the study

The design as a retrospective study included the fact that MRA was only performed in selected cases. We therefore did not show comparisons between duplex ultrasound and MRA.

In this study, we used TCD for the classification of collateral patterns. Data concerning cerebral stenosis are not included. Even if collateral blood flow can be detected by TCD, it is impossible to evaluate the collateral volume capacity. The functional assessment of collateral function is different compared to anatomical investigations, which might explain the finding that we observed a lack of collaterals to any MCA more frequently than that reported in anatomical studies (Lippert & Pabst, 1985).

## Conclusion

Transcranial Doppler helps to determine whether an ICA stenosis is of hemodynamic significance and to assess collateral patterns.

Established collateral blood flow will help to identify patients with ≥70% (ECST) carotid artery disease. TCD might be of value when flow velocity criteria combined with plaque assessment by DUS are inclusive. Other diagnostic methods may also be considered.
